# Supplementary material for: Impact of bimetallic interface design on heat generation in plasmonic Au/Pd nanostructures studied by single-particle thermometry
Source: Nat Commun. 2023 Jun 27;14:3813. doi: 10.1038/s41467-023-38982-9 (PMC10300195; doi:10.1038/s41467-023-38982-9)
Supplement: Supplementary file 1 — Supplementary Information [file 41467_2023_38982_MOESM1_ESM.pdf]

Supplementary Information for

Impact of bimetallic interface design on heat generation in plasmonic Au/Pd nanostructures studied by single-particle thermometry

Julian Gargiulo*, Matias Herran, Ianina L. Violi, Ana Sousa-Castillo, Luciana P. Martinez, Simone Ezendam, Mariano Barella, Helene Giesler, Roland Grzeschik, Sebastian Schlücker, Stefan A. Maier, Fernando D. Stefani, Emiliano Cortes*

*Corresponding authors. Email: jgargiulo@unsam.edu.ar; emiliano.cortes@lmu.de

Table of contents:

Title of the Supplementary Note.	Page
1. Characterization of the Pd shells of Au@Pd CS-NPs.	2
2. Numerical calculation of absorption, scattering and extinction cross sections.	4
3. Stability of the NPs under high irradiances.	6
4 Scattering measurements at the single particle level.	7
5. Photoluminescence at the single particle level.	10
6. Photothermal measurement.	11
7. Temperature modelling of Au@Pd CS-NPs.	12
8. Comparison between lattice and electronic temperatures	14
9. The image method	15
10. Temperature Simulations using COMSOL Multiphysics	17
11. Characterization of Au60-Pd-sat NPs.	19
12. The gap size between the palladium satellites and the gold surface.	22
13. Calculation of absorption efficiencies.	24
14. Photothermal response of alternative materials.	24
15. Calibration for Inductively coupled plasma – atomic emission spectroscopy.	25

Supplementary Note 1. Characterization of the Pd shells of Au@Pd CS-NPs.

The thicknesses of the Pd shells were calculated in two ways. i) From Transmission Electron Microscopy (TEM) images and ii) Using Inductive Coupled Plasma Atomic Emission Spectroscopy (ICP-AES).

1. Transmission Electron Microscopy Images.

The area A of each NP was measured, and a characteristic diameter was calculated as $d = 2\sqrt{\frac{A}{\pi}}$. 80 NPs were measured for each colloid, and the median $\bar{d}$ and standard deviation σ_{d} of the distributions were calculated. The standard deviations were in the range between 1 nm and 2 nm for all the colloids studied in the manuscript. The standard errors of the means were calculated as $\sigma_{\bar{\mathrm{d}}} = \frac{\sigma_{\mathrm{d}}}{\sqrt{N}}$ with N the sample size.

For Core Shells, the thickness t of the Pd shell was estimated as $t = \bar{d}_{\mathrm{CS}} - \bar{d}_{\mathrm{C}}$, where $\bar{d}_{\mathrm{CS}}$ and $\bar{d}_{\mathrm{C}}$ are the median diameters of the Core Shell and Au Cores respectively. The error in the thickness t is the addition of the absolute standard errors of the median diameters.

TEM images and size distributions for Au67 NS, Au67@Pd2 and Au67@Pd4 are shown in Figure 1 of the main manuscript. Supplementary Figure 1 shows TEM images and size distributions for Au60 NS, and Au60@Pd2.

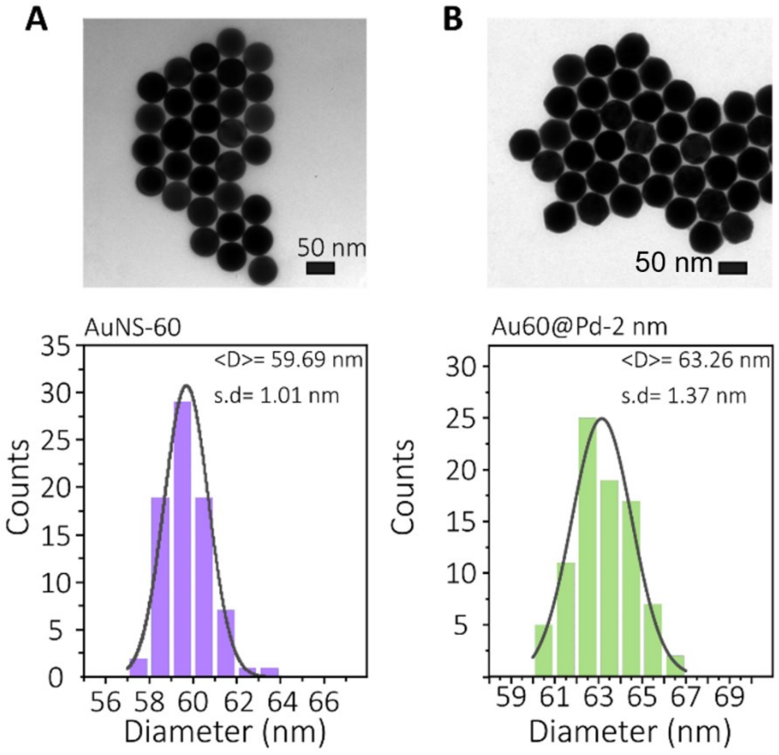

Supplementary Figure 1. Size Characterization of Core-shell nanoparticles. Top: TEM image. Bottom: corresponding size distribution. **(a)** Au NS-60. **(b)** Au60@Pd.

2. Inductive Coupled Plasma Atomic Emission Spectroscopy (ICP-AES).
Additional characterization of the Core Shell NPs was performed using (ICP-AES). This technique allows the quantification of the ratio between the Au and Pd mass (see section S15 for details). Using this information, knowing the size of the Au cores, and assuming a perfectly spherical geometry for the cores and the CS, the thickness of the Pd shell can be estimated as

$$t = \sqrt[3]{\frac{3}{4\pi}(V_C + V_S)} - r_C = r_C\left(\sqrt[3]{1 + \frac{\rho_C}{\rho_S}\frac{M_S}{M_C}} - 1\right)$$

With $V_C = \frac{4}{3}\pi\, r_C^3$, $V_S = V_C \frac{\rho_C}{\rho_S}\frac{M_S}{M_C}$. V is the volume, r is the radius, M is the mass and ρ is the density. Subscripts C and S refer to the core and the shell, respectively. The ratios $\frac{M_S}{M_C}$ are measured using ICP-AES.

The obtained results using both methods are summarized in Suppl. Table 1.

	Core Size (TEM)	Pd/Au $\frac{M_S}{M_C}$	Pd Thickness (ICP-AES)	Pd Thickness (TEM)
Au67@Pd2	(66.6 ± 0.2) nm	(0.105 ± 0.005)	(1.8 ± 0.4) nm	(2.4 ± 0.3) nm
Au67@Pd4	(66.6 ± 0.2) nm	(0.21 ± 0.01)	(3.4 ± 0.4) nm	(3.6 ± 0.3) nm
Au60@Pd2	(59.7 ± 0.2) nm	(0.073 ± 0.004)	(1.1 ± 0.4) nm	(1.8 ± 0.3) nm

Supplementary Table 1. Summary of size characterization of CS-NP.

Crystallinity of the Pd shell was studied Scanning Transmission Electron Microscopy (STEM) in High Angle Annular Darkfield (HAADF) mode and in Bright Field mode. The Supplementary Figure 2 shows two images corresponding to the Au67@Pd4 system. The images indicate that growth of Pd on the Au core is crystalline.

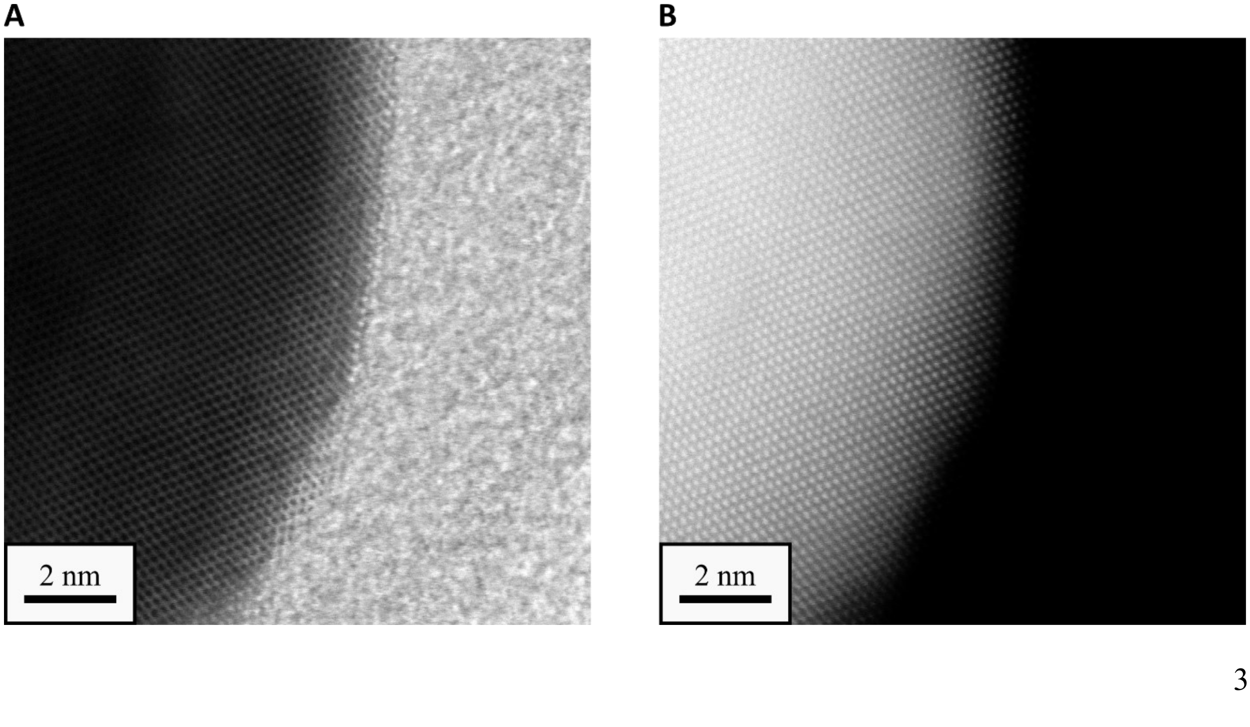

Supplementary Figure 2. Crystallinity of Core-shell nanoparticles. Scanning Transmission Electron Microscopy in High Angle Annular Darkfield **(a)** and Bright Field **(b)** mode. Images correspond to a Au67@Pd4 CS-NP.

Supplementary Note 2. Numerical calculation of absorption, scattering and extinction cross sections.

Optical properties of the core-shell bimetallic nanoparticles were investigated using FDTD simulations (ANSYS Lumerical Software ULC, Release 2022 R1, Version 8.27.2898).
The dimensions of the spherical Au (Johnson & Christy) cores were chosen as measured from the TEM images (diameters of 60 and 67 nm). The Pd shell was simulated at different thicknesses ranging from 1 nm to 6 nm by a Pd (Palik) with a lower mesh order than the Au. To simulate an aqueous medium the surrounding medium was set to a refractive index of 1.333. The substrate, when included, was simulated as a rectangular prism consisting of Glass (Palik). To calculate absorption and scattering cross-sections a TFSF source was used. The injection was simulated along the z-axis. The absorption cross-section is calculated using an analysis group inside the TFSF source. The analysis group consists of six 2D monitors that form a closed box and measure the net power flowing in/out of the box. Similarly, the scattering cross-section is calculated by an analysis group outside the TFSF source.
Since this simulation has a plane of symmetry in x and in y dimension, the simulation time was reduced by setting the x min boundary condition to anti-symmetric and the y min boundary condition to symmetric.
A mesh override region with d = 0.5 nm until 30 nm around the nanoparticle in all dimensions was used.

Influence of the substrate on the absorption cross sections
To investigate the role of the substrate on the absorption cross section values, three variations of the particle-substrate distance were performed. The different core-shell particles were put either put directly on the substrate, embedded 2 nm or at a height 2 nm from the substrate. The figure below shows the results for the 67 nm Au cores. The simulations show that the inclusion of the substrate has an effect of max. ≈ 3 % on the absorption cross section and the effect of the variation of the substrate height is even smaller. The table below shows the calculated absorption cross sections at 531.4 nm.

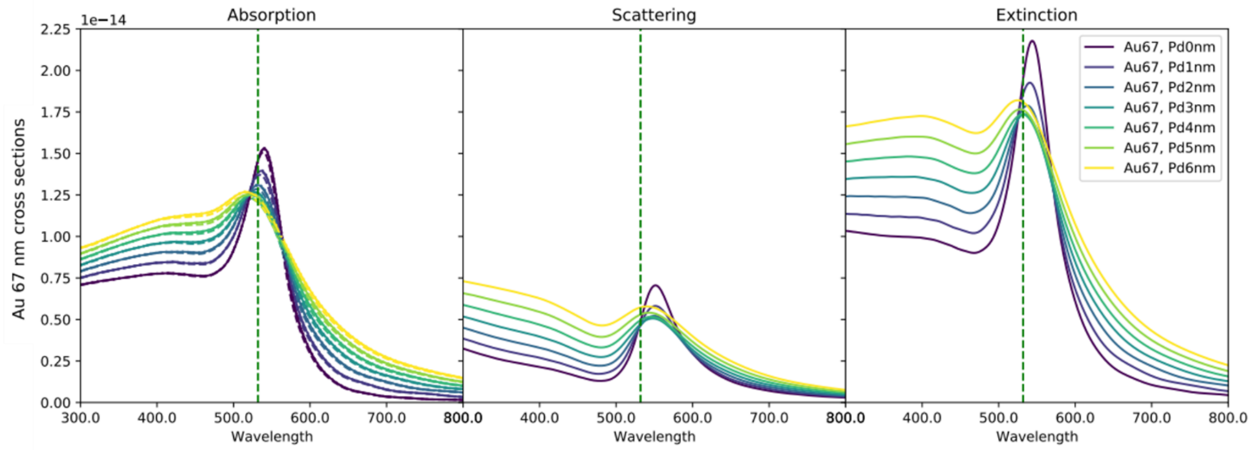

Supplementary Figure 3. Au67@Pd absorption, scattering and extinction cross sections (m^2) for different interfaces between the NP and the glass substrate. The line style is as follows. Dashed: no substrate, solid: substrate at 0 nm distance from the NP, dotted: NP embedded into substrate by 2 nm distance, dash-dotted: 2 nm distance between NP and substrate. The green vertical line indicates the illumination wavelength. The three plots share the y-axis.

	No substrate	With substrate distance 0 nm		With substrate distance 2 nm		With substrate embedded 2 nm	
Au67	1.4469	1.4490	0.15%	1.4494	0.17%	1.4490	0.14%
Au67_Pd1nm	1.3640	1.3814	1.28%	1.3791	1.11%	1.3843	1.49%
Au67_Pd2nm	1.2855	1.3089	1.82%	1.3056	1.56%	1.3129	2.13%
Au67_Pd3nm	1.2380	1.2637	2.08%	1.2601	1.79%	1.2681	2.43%
Au67_Pd4nm	1.2137	1.2407	2.23%	1.2371	1.93%	1.2452	2.59%
Au67_Pd5nm	1.2060	1.2338	2.31%	1.2302	2.01%	1.2382	2.67%
Au67_Pd6nm	1.2112	1.2393	2.32%	1.2358	2.04%	1.2436	2.68%

Supplementary Table 2. Calculated absorption cross sections at 531.4 nm (10^{-14} m^2) and its dependance with the presence of a substrate at different distances.

<u>Influence of the core size on the absorption cross sections</u>
The account for the heterogeneity of the Au core size in the 67 nm sample, absorption cross sections were also simulated for 63.6 nm and 71 nm cores. The table below shows the obtained values. As the change is much larger than for the inclusion/variation of the substrate, these values were used as the min/max values of the absorption cross sections in the "predicted range" in Figure 3.

Pd Shell	Absorption cross section at 531.4 nm (10^{-14} m^2), all on substrate (distance 0 nm)	
	Core size 63.6 nm Au	Core size 71 nm Au
Pd 0 nm	1.3268	1.5985
Pd 1 nm	1.2391	1.5537
Pd 2 nm	1.1594	1.4904
Pd 3 nm	1.1131	1.4457
Pd 4 nm	1.0909	1.4200
Pd 5 nm	1.0866	1.4104
Pd 6 nm	1.0947	1.4122

Supplementary Table 3 - Influence of the Au core size on absorption cross sections

Supplementary Note 3. Stability of the NPs under high irradiances.

In order to test the stability of the NPs under the high irradiances used for printing, a set of NPs was illuminated at $I = I_{\text{Printing}}$ and $I = 2I_{\text{Printing}}$ for 2 minutes. It must be noted that 2 minutes is much longer than the printing time that is typically some milliseconds.[1] In addition, irradiances used for photothermal characterizations were always lower than the printing one.
The scattering spectrum of each NP before and after irradiation was compared. Supplementary Figure 4A shows a dark field image of 10 Au60@2Pd CS-NPs before and after 2 minutes at $I = I_{\text{Printing}}$. The average scattering spectra is mostly unchanged, as shown in the upper plot of Supplementary Figure 4B, indicating that the CS-NPs are stable under this irradiance.
Then, another set of 10 CS-NPs was irradiated at twice the printing irradiance. In this case, the scattering spectra significantly changes, as evident both from the dark field images shown at Supplementary Figure 4C and the average spectra shown in the lower plot of Supplementary Figure 4B.

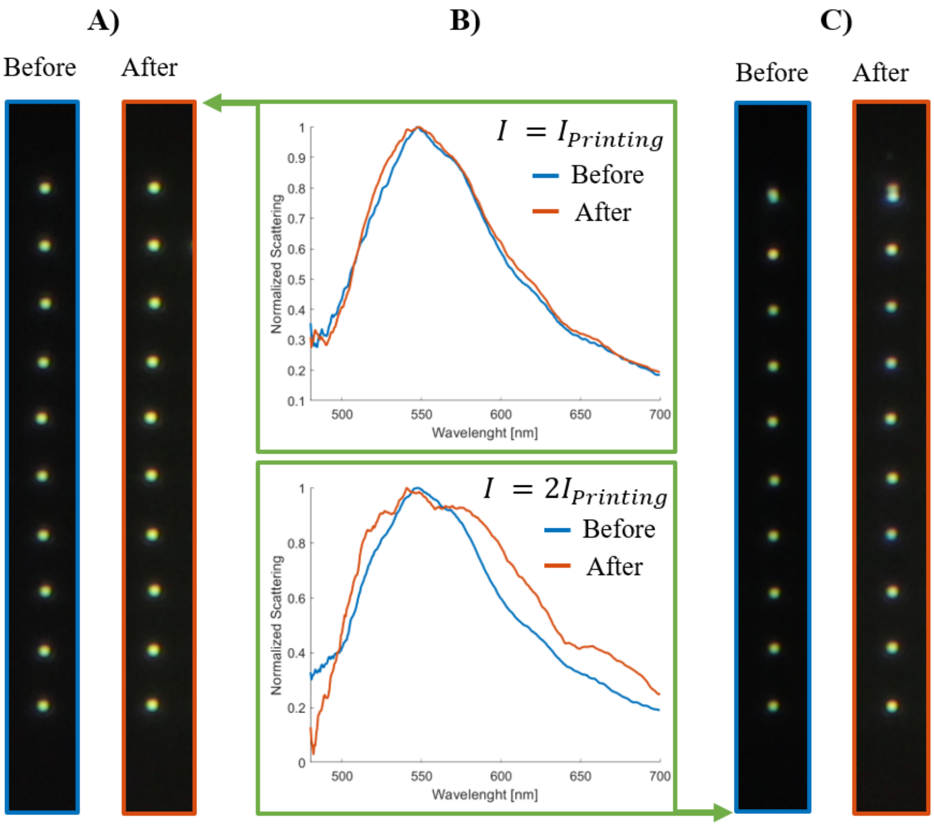

Supplementary Figure 4. Stability Test. A) Dark-field image of 10 Au60@2Pd before and after irradiation at the printing irradiance for 2 minutes. B) Average scattering spectra before and after irradiation. C) Dark-field image of 10 Au60@2Pd before and after irradiation at the twice the printing irradiance for 2 minutes.

<u>Supplementary Note 4 Scattering measurements at the single particle level.</u>

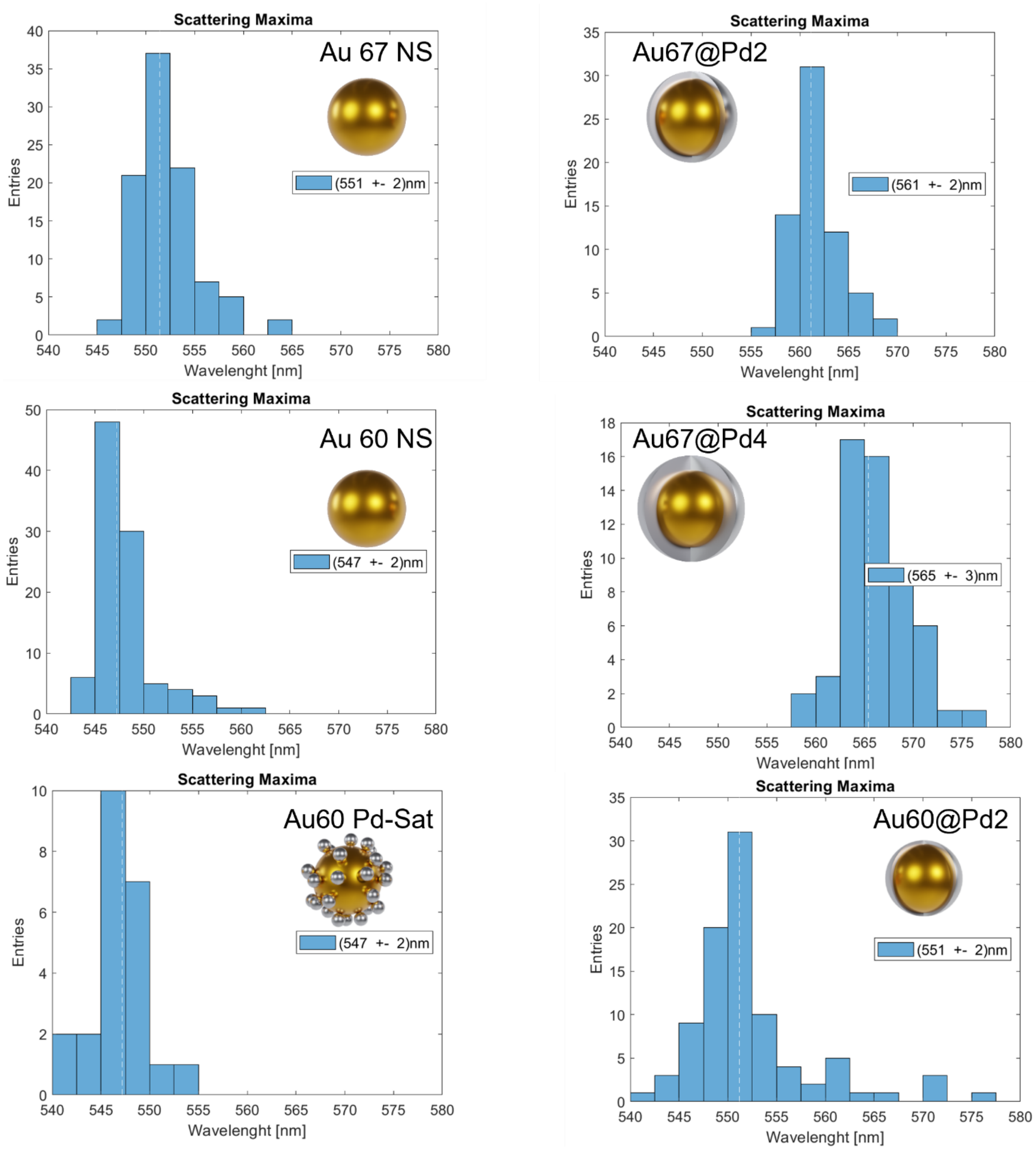

Supplementary Figure 5. Histograms of the maximum wavelength of the single NP scattering spectra.

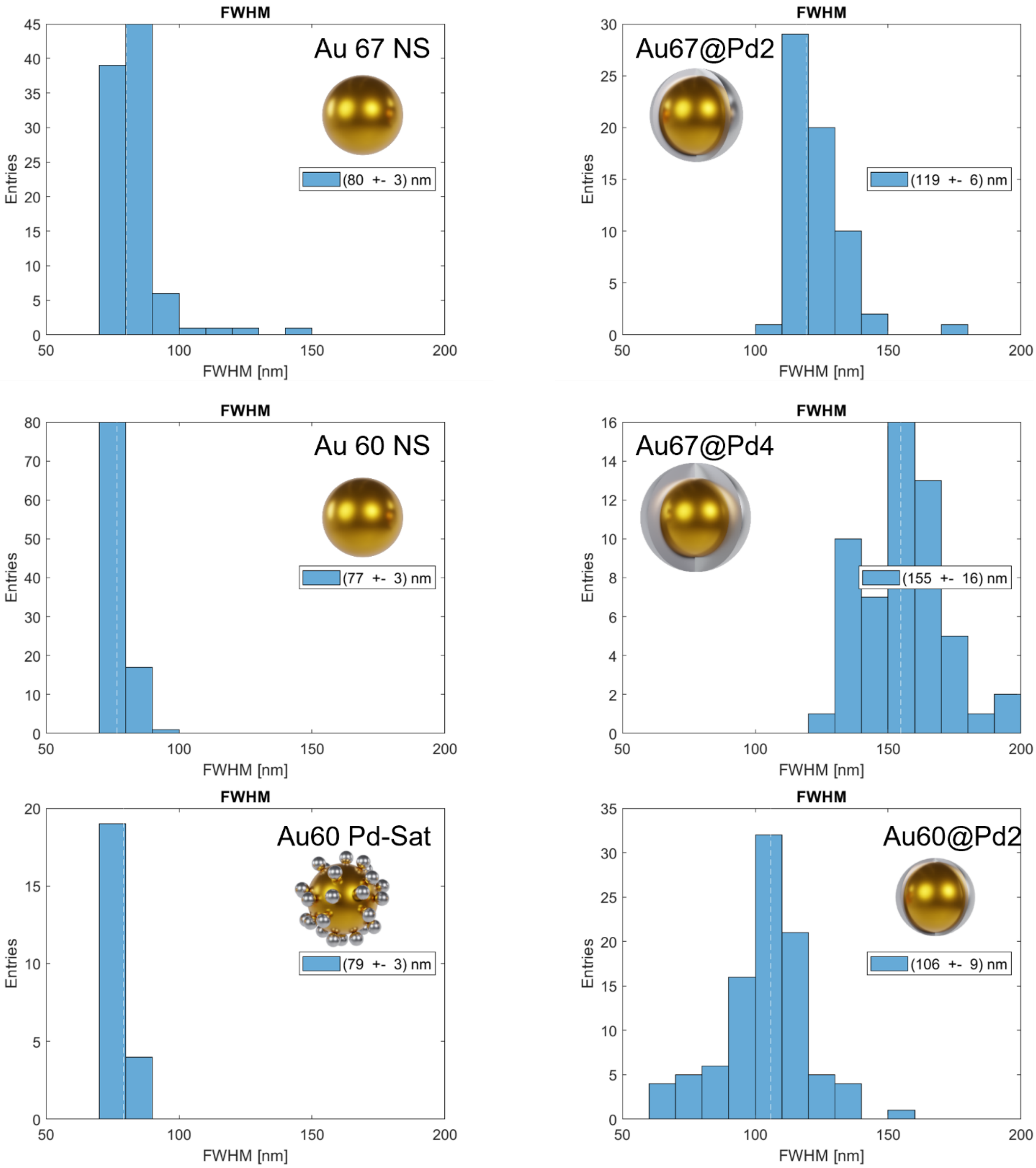

Supplementary Figure 6. Histograms of FWHM of the single NP scattering spectra.

Supplementary Note 5. Photoluminescence at the single particle level.

Supplementary Figure 7 shows the integrated PL Stokes counts per second, divided by the excitation irradiance. Calculated using $\frac{\int_{543}^{\infty} \mathrm{PL}(\lambda)\ d\lambda}{[t]\ I_{\mathrm{exc}}}$, where [t] is the integration time.

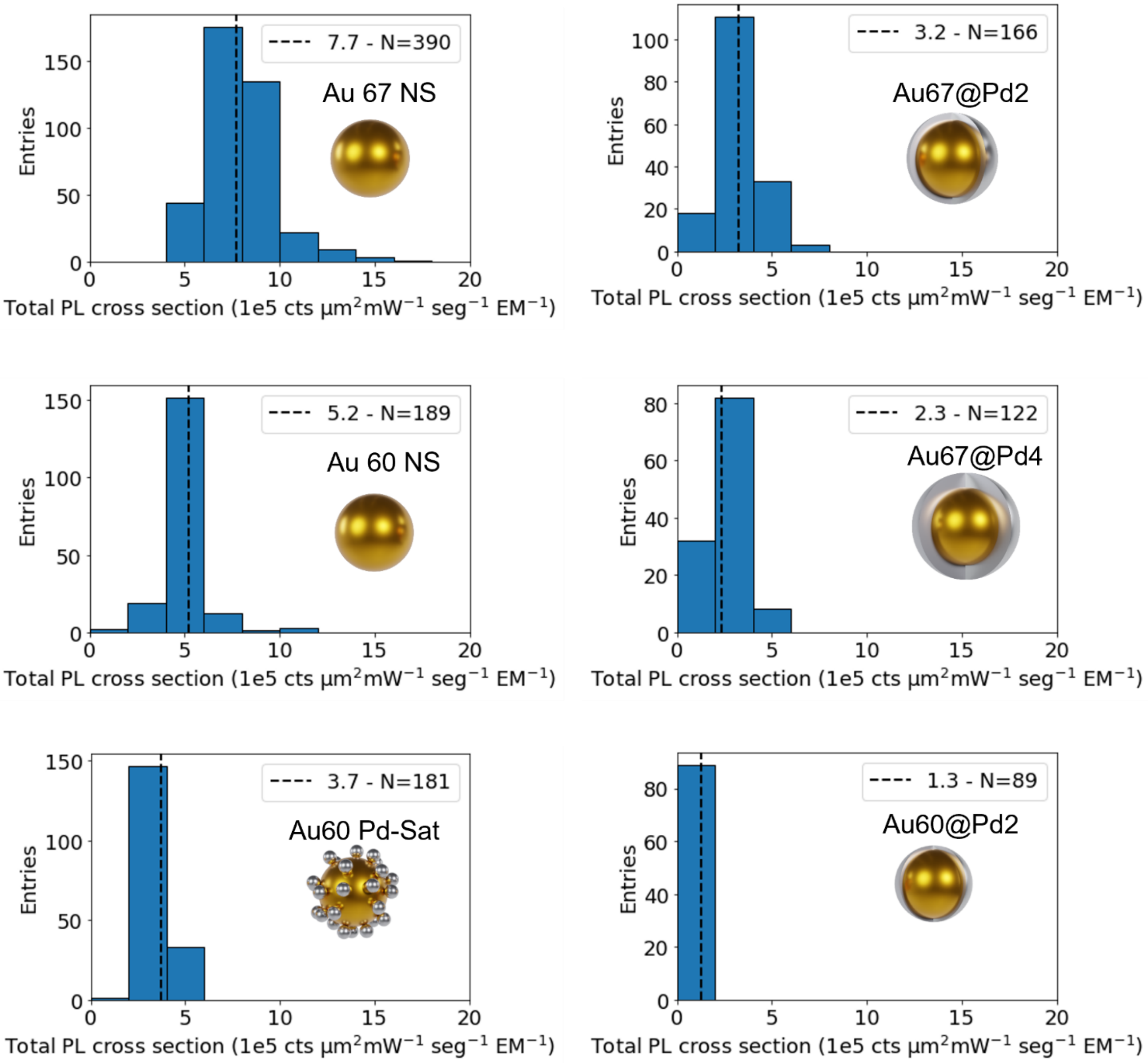

Supplementary Figure 7. Histograms of PL Stokes emission.

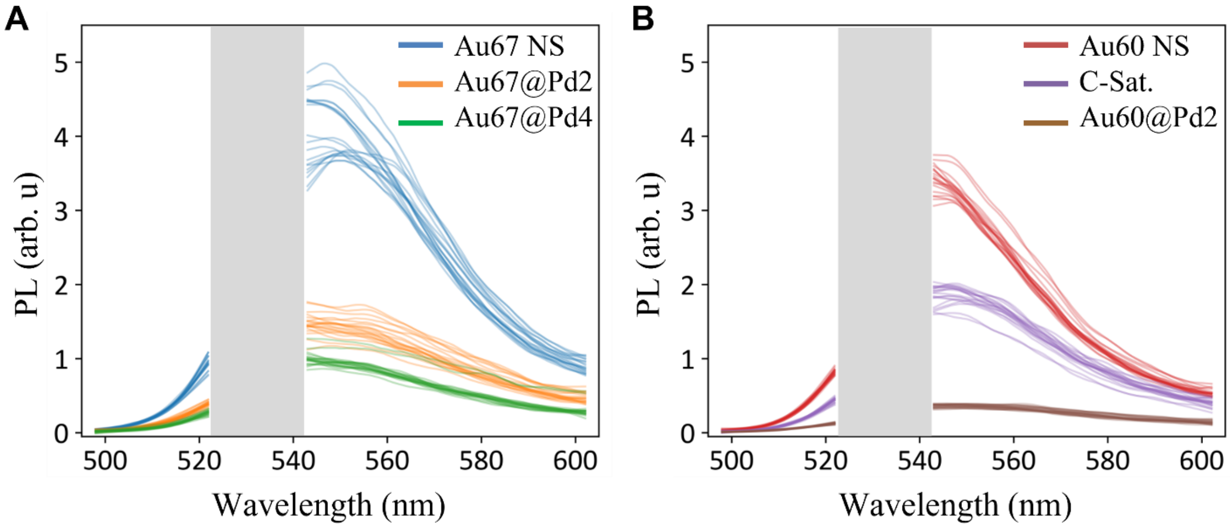

Supplementary Figure 8. Single particle PL emission spectra, excited with 1 mWμm^{-2} of laser light at 532 nm. Grey band with no data corresponds to the laser rejection filter.

<u>Supplementary Note 6. Photothermal measurement.</u>

The hyperspectral confocal image provides 10x10 PL spectra that are grouped and subsequently averaged considering similar irradiance values. The sorting criterion is the excitation irradiance I_i^{exc}. Supplementary Figure 9B shows the hyperspectral image for an Au60@Pd2 CS-NPs after being binned into 10 irradiance values. Supplementary Figure 9C shows the PL average spectra of each bin. Then, all possible ratios between binned PL spectra are calculated. Supplementary Figure 9D shows calculated ratios between Stokes PL spectra, named $Q_{i,j}^{\text{S}}$. Since PL Stokes emission is linear with excitation irradiance, $Q_{i,j}^{\text{S}} = \frac{I_i^{\text{exc}}}{I_j^{\text{exc}}}$. Supplementary Figure 9E shows a few calculated ratios between AS PL emission spectra, named $Q_{i,j}^{\text{AS}}$. These ratios are fitted with the expression $Q_{i,j}^{\text{AS}}(\lambda) = Q_{i,j}^{\text{S}} \frac{e^{\frac{E(\lambda)-E(\lambda_{\text{exc}})}{k_{\text{B}}\left[T_0+\beta_{i,j} I_j^{\text{exc}}\right]}}-1}{e^{\frac{E(\lambda)-E(\lambda_{\text{exc}})}{k_{\text{B}}\left[T_0+\beta_{i,j} I_i^{\text{exc}}\right]}}-1}$ to extract the photothermal coefficient $\beta_{i,j}$. Finally, all obtained photothermal coefficient $\beta_{i,j}$ are averaged to obtain a single β for the scanned NP.

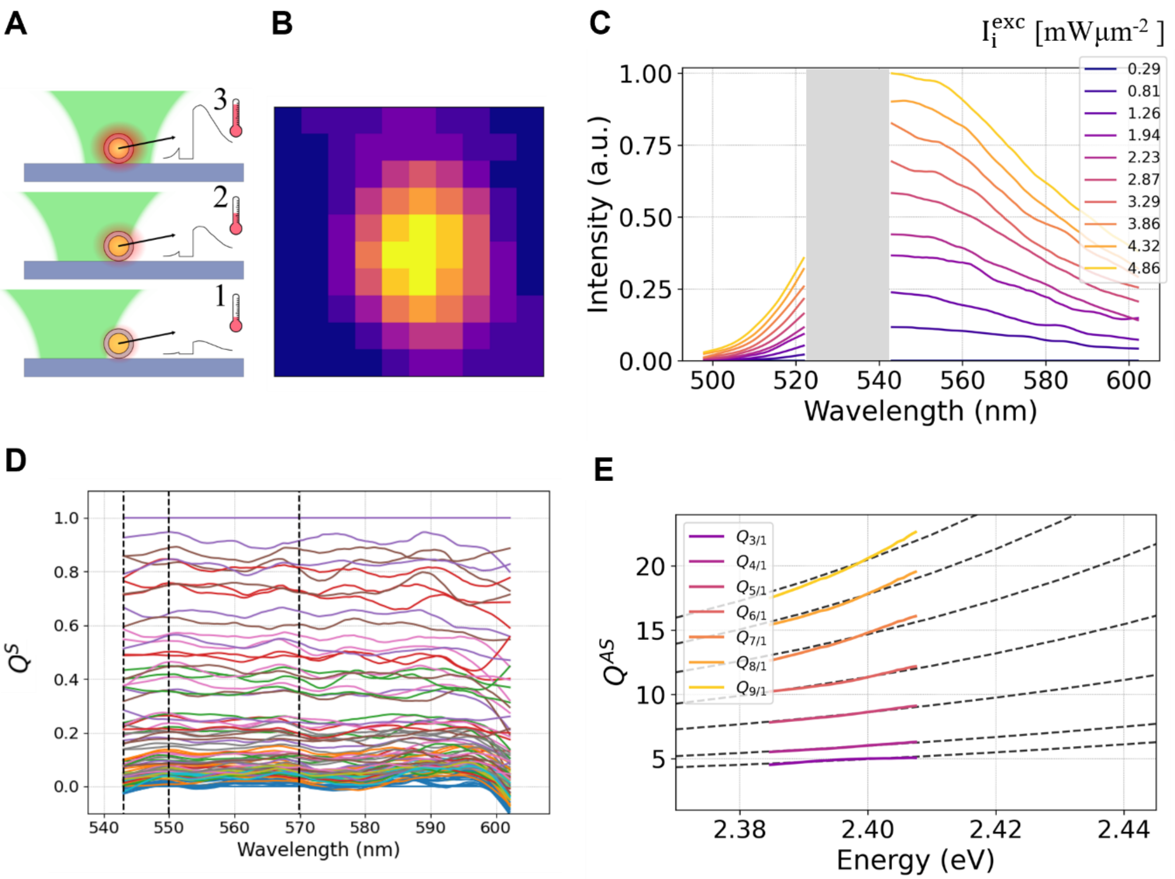

Supplementary Figure 9. Hyperspectral thermometry. A) Illustration of the method for hyperspectral AS thermometry. Heating and PL excitation are performed simultaneously with the same beam. The laser is raster scanned, changing the relative position between the NP and the beam. For each relative position (exemplified with numbers 1,2,3), the excitation irradiance seen by the NP is different, and hence, it reaches a different temperature. At each position, a PL spectrum is acquired. B) Hyperspectral PL emission image, binned according to the excitation irradiance. C) Average PL emission spectra of each bin. The estimated excitation irradiance is indicated in the label. D) Example of ratios between PL Stokes spectra used to calculate $Q_{i,j}^{S}$. E) Ratio between PL AS Stokes spectra. Experimental data are the colored solid lines, while dashed black lines indicate the fits with $Q_{i,j}^{AS}(\lambda)$.

Supplementary Note 7. Temperature modelling of Au@Pd CS-NPs.

The temperature field $T(r)$ of core@shell NPs can be calculated using the heat diffusion equation that for a system with radial symmetry in the steady states reads

$$-\kappa \frac{1}{r^2} \partial r (r^2 \partial r T) = q \quad (1)$$

Where κ is the thermal conductivity and q is the heat power density. The solution for system with a core material 1, shell material 2 immersed in a medium material 3 is:

$r < b$
$T(r) = \frac{-q_1}{6\kappa_1} r^2 + \frac{c_0}{r} + c_1$

$b < r < a$ (2)
$(r) = \frac{-q_2}{6\kappa_2} r^2 + \frac{c_2}{r} + c_3$
$r > a$
$T(r) = \frac{c_4}{r} + c_5$

where c_0 to c_5 are integration constants. $c_5 = T(\infty) = T_0$ with T_0 the room temperature. Non divergence at $T(0)$leads to $c_0 = 0$. The others four constant can be obtained from the following boundary conditions.

1. Energy conservations demands that the integrated heat flux J crossing interface 2-3 should be equal to the absorbed heat Q by the NP.

   $4\pi a^2 |J(\mathrm{a}^+)| = Q$

   Using $J(r) = \kappa \nabla T(r)$ leads to

   $c_4 = \frac{Q}{4\pi\kappa_3}$

2. Heat flux conservation at the interface 2-3

   $|J(\mathrm{a}^-)| = |J(\mathrm{a}^+)|$

   Leads to

   $c_2 = \frac{\kappa_3}{\kappa_2} c_4 - \frac{q_{2a^3}}{3\kappa_2}$

3. Temperature boundary condition at the interface 2-3

   $T(\mathrm{a}^-) - T(\mathrm{a}^+) = |J(\mathrm{a}^+)| R^{\mathrm{th}}_{2-3}$

   R^{th}_{2-3} is the Kapitza interfacial thermal resistance between materials 2 and 3. This leads to

   $c_3 = \frac{\kappa_3 R^{\mathrm{th}}_{2-3} c_4}{a^2} + \frac{c_4}{a} + \frac{q_{2a^2}}{6\kappa_2} - \frac{c_2}{a} + T_0$

4. Temperature boundary condition at the interface 1-2

   $T(b^-) - T(b^+) = |J(b)| R^{\mathrm{th}}_{1-2}$

Using $\boldsymbol{4\pi b^2 J(b)} = \frac{4}{3}\boldsymbol{\pi b^3} q_1$

Leads to

$$c_1 = \frac{1}{3} q_1 \boldsymbol{b} R^{\mathrm{th}}_{1-2} + c_3 + \frac{c_2}{b} + \frac{q_{1b^2}}{6\kappa_1} - \frac{q_{2b^2}}{6\kappa_2}$$

Supplementary Note 8. Comparison between lattice and electronic temperatures

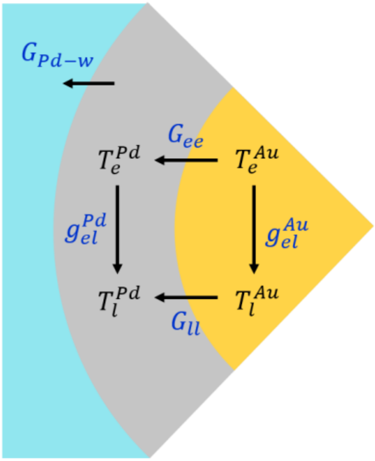

Supplementary Figure 10. Visual representation of the parameters used in the 4 Temperature model.

Due to the high thermal conductivity of Au and Pd, temperature variations within each material can be neglected. Thus, the system can be described using four relevant temperatures, as shown in Supplementary Figure 10. These are the Au electronic $T_{\mathrm{e}}^{\mathrm{Au}}$ and $T_{\mathrm{l}}^{\mathrm{Au}}$ lattice temperatures, and the Pd electronic T_e^{Pd} and T_l^{Pd} lattice temperatures. Four temperature differences are defined. $\Delta\mathrm{T_e} = T_{\mathrm{e}}^{\mathrm{Au}} - T_{\mathrm{e}}^{\mathrm{Pd}}$, $\Delta\mathrm{T_{Au}} = T_{\mathrm{e}}^{\mathrm{Au}} - T_{\mathrm{l}}^{\mathrm{Au}}$, $\Delta\mathrm{T_{Pd}} = T_{\mathrm{e}}^{\mathrm{Pd}} - T_{\mathrm{l}}^{\mathrm{Pd}}$, $\Delta\mathrm{T_l} = T_{\mathrm{l}}^{\mathrm{Au}} - T_{\mathrm{l}}^{\mathrm{Pd}}$. Total absorbed heat is Q, distributed in the absorbed heat by Au electrons $Q_1 = \chi_1 Q$ and Pd electrons $Q_2 = \chi_2 Q$. Energy conservation of each subsystem reads:

$$\begin{aligned}
Q_1 &= V^{\mathrm{Au}} g_{\mathrm{e-l}}^{\mathrm{Au}} \Delta\mathrm{T_{Au}} + S G_{\mathrm{ee}}^{\mathrm{AuPd}} \Delta\mathrm{T_e} \\
0 &= -V^{\mathrm{Au}} g_{\mathrm{e-l}}^{\mathrm{Au}} \Delta\mathrm{T_{Au}} + S G_{\mathrm{l}}^{\mathrm{AuPd}} \Delta\mathrm{T_l} \\
Q_2 &= -S G_{\mathrm{ee}}^{\mathrm{AuPd}} \Delta\mathrm{T_e} + V^{\mathrm{Pd}} g_{\mathrm{e-l}}^{\mathrm{Pd}} \Delta\mathrm{T_{Pd}} \\
0 &= -\Delta\mathrm{T_{Au}} + \Delta\mathrm{T_e} - \Delta\mathrm{T_l} + \Delta\mathrm{T_{Pd}}
\end{aligned} \tag{3}$$

where V^{Au} and V^{Pd} are the volumes of each material and S the area of their interface. $G_{\mathrm{ee}}^{\mathrm{AuPd}}$ and $G_{\mathrm{ll}}^{\mathrm{AuPd}}$ are the electronic (lattice) thermal conductance of the interface, respectively. $g_{\mathrm{e-l}}^{\mathrm{Au}}$ and $g_{\mathrm{e-l}}^{\mathrm{Pd}}$ are the electron-phonon coupling of Au and Pd, respectively. For a fixed Q, these are four equation with four unkown values $\Delta\mathrm{T} = (\Delta\mathrm{T_{Au}}, \Delta\mathrm{T_e}, \Delta\mathrm{T_l}, \Delta\mathrm{T_{Pd}})$. To provide an adimensional number

independent of Q, the ratio $\frac{\Delta T}{\Delta T_w}$ was calculated, with $\Delta T_w = T(a^+) - T_0 = \frac{Q}{4\pi\kappa_3 a}$. This allows the estimation of the temperature differences as a fraction of the overall NP temperature increase over room temperature. The calculation requires several thermodynamical constants for which accurate experimental values are scarce or too disperse. For this reason, upper and lower bounds for $\frac{\Delta T}{\Delta T_w}$ were calculated using the maximum and minimum values found for each parameter, as described in Suppl. Table 4. $G_{\mathrm{Au-Pd}}^{\mathrm{e-e}}$ was calculated from Wiedemann-Franz law $G = \frac{LT}{(AR)}$, $L = 2.45 \times 10^{-8} \Omega \mathrm{WK}^{-2}$ and (AR) the specific electrical resistance of the interface.[2] The value of $(\mathrm{AR})_{\mathrm{Au-Pd}} = 0.23 \times 10^{-15} \Omega\ \mathrm{m}^{-2}$ was taken from Galinon *et. al.*[3].

Then, $\frac{\Delta T}{\Delta T_w}$ was calculated for all the Au@Pd CS-NPs employed in this work. The largest values were $\frac{\Delta T}{\Delta T_w} = (0.012,0.0003,0.006,0.018)$ for Au60@Pd2, $\frac{\Delta T}{\Delta T_w} = (0.01,0.0003,0.006,0.016)$ for Au67@Pd2 and $\frac{\Delta T}{\Delta T_w} = (0.006,0.0003,0.003,0.01)$ for Au67@Pd4. This means that the upper bound for the differences between the electronic and lattice temperatures are 1.2% for Au and 1,8% for Pd.

Parameter	Min Value	Reference	Max Value	Reference
$g_{\mathrm{e-l}}^{\mathrm{Au}}$	$2.2\times 10^{16}\ \mathrm{W\,m^{-3}K^{-1}}$	4	$3\times 10^{16}\ \mathrm{W\,m^{-3}K^{-1}}$	5
$g_{\mathrm{e-l}}^{\mathrm{Pd}}$	$2.5\times 10^{16}\ \mathrm{W\,m^{-3}K^{-1}}$	6	$87\times 10^{16}\ \mathrm{W\,m^{-3}K^{-1}}$	7
(χ_1, χ_2)	(0.95,0.05)		(0.05,0.95)	
$G_{\mathrm{Au-Pd}}^{\mathrm{e-e}}$	$37 \times 10^{9}\mathrm{W\,m^{-2}K^{-1}}$ [2]			
$G_{\mathrm{ll}}^{\mathrm{AuPd}}$	$0.6 \times 10^{9}\mathrm{W\,m^{-2}K^{-1}}$ [8]			

Supplementary Table 4. Thermodynamic constants for the 4-temperature model.

Supplementary Note 9. The image method

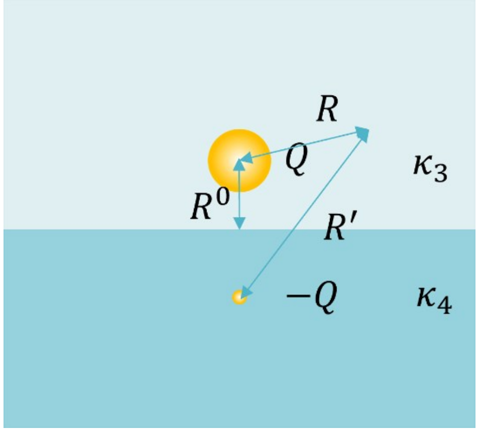

Supplementary Figure 11. Image method. Schematic of the system composed of a NP in a media of thermal conductivity κ_3 close to a substrate with thermal conductivity κ_4.

The problem of thermal conduction close to a substrate is equivalent to solving the electrostatic problem of charge sources in front of an infinite planar conductor because both are governed by the Laplace equation. In analogy with the image method of electrostatic, the effect of the substrate can be calculated by considering a virtual charge image on the substrate side. For a spherical NP located at the position $(0,0,R^0)$ with total absorbed heat Q, the virtual image is a point source $-Q$ located at $(0,0,-R^0)$.
The temperature *T* on a position *R* outside the NP and the upper medium is the addition of the real and the virtual heat sources.

$$T(R) = \frac{Q}{4\pi\kappa_3}\left(\frac{1}{|R|} - \left(\frac{\kappa_4-\kappa_3}{\kappa_4+\kappa_3}\right)\frac{1}{|R'|}\right) \quad (4)$$

Where *R* are the distances to the real and virtual sources, respectively. The average surface temperature $\langle T^S \rangle$ on a spherical NP of radius a is given by the surface integral

$$\langle T^{\mathrm{S}} \rangle = \frac{1}{4\pi a^2} \oiint T(a,\theta,\pi) sin\theta \;\; a^2 \, d\theta \, d\varphi \quad (5)$$

Where θ is the polar angle and φ is the azimuthal angle. The problem has symmetry in φ. Therefore

$$\langle T^{\mathrm{S}} \rangle = \frac{1}{2}\int_0^{\pi} \frac{Q}{4\pi\kappa_3}\left(\frac{1}{a} - \left(\frac{\kappa_4-\kappa_3}{\kappa_4+\kappa_3}\right)\frac{1}{|R'(\theta)|}\right) sin\theta d\theta \quad (6)$$

Using the law of cosines, it can be shown that $|\mathrm{R}'(\theta)| = \sqrt{|2R^0|^2 + |a|^2 - 2a|2R^0|\cos(\pi-\theta)}$.

If the NP is in contact with the substrate, $|R^0| = a$.

$$\langle T^{\mathrm{S}} \rangle = \frac{1}{2}\frac{Q}{4\pi\kappa_3}\int_0^{\pi}\left(\frac{1}{a} - \left(\frac{\kappa_4-\kappa_3}{\kappa_4+\kappa_3}\right)\frac{1}{\sqrt{5a^2+4a^2\cos(\theta)}}\right) sin\theta d\theta \quad (7)$$

$$\langle T^{\mathrm{S}} \rangle = \frac{Q}{4\pi a\kappa_3}\left(1 - \frac{1}{2}\left(\frac{\kappa_4-\kappa_3}{\kappa_4+\kappa_3}\right)\right) = f\,\frac{Q}{4\pi a\kappa_3} \quad (8)$$

The result is the well-known expression for an isolated NP in a homogeneous media $\frac{Q}{4\pi a\kappa_3}$, multiplied by a factor $f = 1 - \frac{1}{2}\left(\frac{\kappa_4-\kappa_3}{\kappa_4+\kappa_3}\right)$. For a NP immersed in water on a glass substrate, the value of f is 0.875. Remarkably, a temperature simulation using COMSOL Multiphysics (See section S10 for details.) gives the value of f=0.878 for a spherical NP in a water environment and touching a glass substrate on a single contact point.

Supplementary Note 10. Temperature Simulations using COMSOL Multiphysics

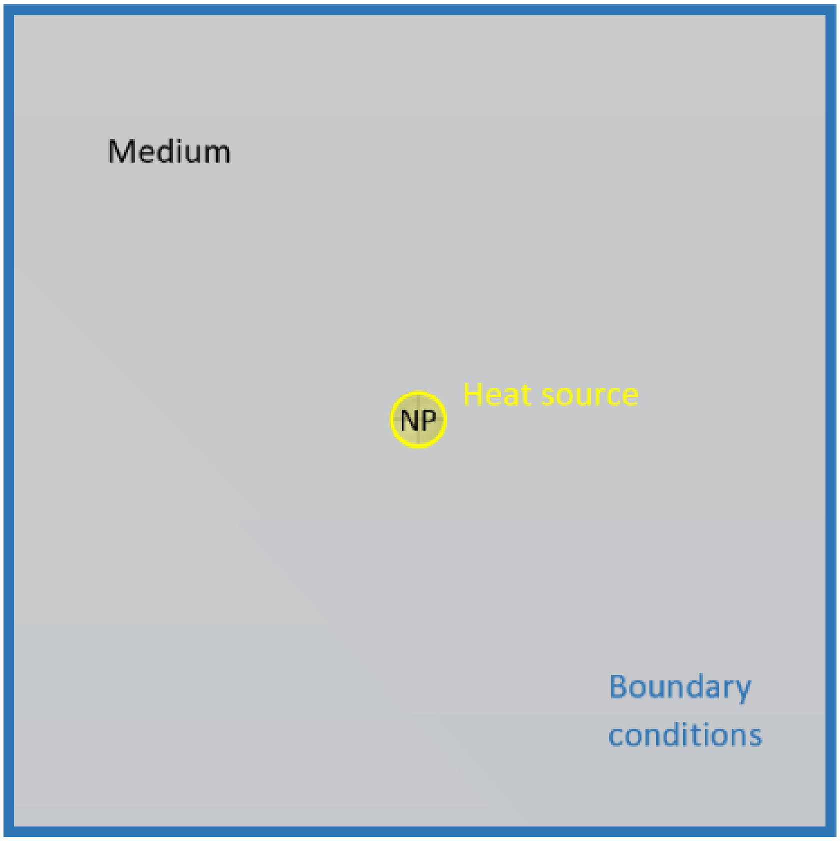

Supplementary Figure 12. 2D view of the 3D simulation in COMSOL Multiphysics. The boundary conditions are applied to the blue boundaries, the NP acts as the heat source.

Medium. Geometry: Cube with dimensions 1000 nm x 1000 nm x 1000 nm. Material: Water, $k = 0.6\ \mathrm{W\ m^{-1}K^{-1}}$
Particle. Geometry: sphere with radius = 33.5 nm. Material: Gold, k = 318 $\mathrm{W\ m^{-1}K^{-1}}$
Physics. Heat transfer in solids with the nanoparticle as an user defined heat source (shown in yellow) with $Q = \frac{\sigma_{\mathrm{abs}} * I}{V}$, where the absorption cross section is $\sigma_{\mathrm{abs}} = 1.4469 * 10^{-14}\ \mathrm{m^2}$ (as calculated using Lumerical), $I = 1\ \mathrm{mW\ \mu m^{-2}}$ and the volume for the Au 67 NS $V = 157479\ \mathrm{nm^3}$. The simulation boundaries (in this case the outer surfaces of the cube of water, shown in blue) and the initial temperatures are set to T0 = 293 K.

Estimation of the role of the substrate in COMSOL Multiphysics:

To simulate the influence of the substrate on the temperature of the NP we slightly modified the geometry of the simulation by splitting the medium into the surrounding medium water and the substrate glass ($k = 1\ \mathrm{W\ m^{-1}\ K^{-1}}$). The two geometries are shown in Supplementary Figure 13. The absorption cross section for the Au NS on the substrate was adjusted to $\sigma_{\mathrm{abs}} = 1.45 * 10^{-14}\ \mathrm{m^2}$, as calculated using Lumerical FDTD. The resulting temperature in the NS on the substrate is 87.8 % of the temperature of the NS in water. Remarkably, this is value is in high

agreement with the obtained using the image method, for a spherical NP in a water environment and touching a glass substrate on a single contact point the value of f=0.875 (see Supplementary Note 9).

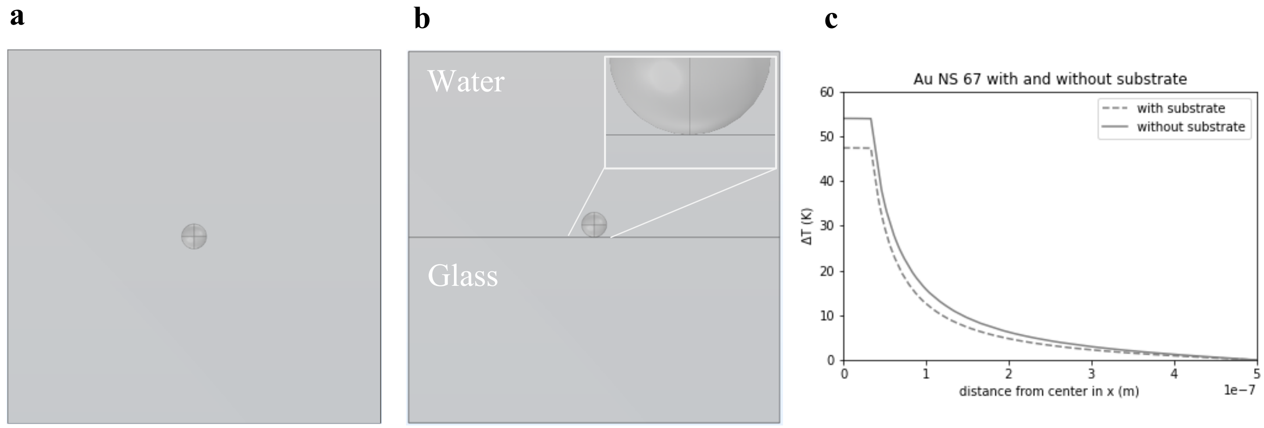

Supplementary Figure 13. Influence of substrate calculated by COMSOL Multiphysics. (a) 2D view of the simulation setup of the NS in water. (b) 2D view of the simulation setup of the NS on a substrate. The inset shows a zoom in, the particle touches the substrate in one point. (c) Resulting temperature profiles from the center of the NS in x direction.

<u>Estimation of the influence of facets in COMSOL Multiphysics</u>

While the measured temperatures of the Au NS fall within the range of our analytical predictions, the analytical model predicts a higher temperature for the core shell particles. Looking closely at the TEM images, the Pd shell does not appear to be perfectly spherical but seems to be slightly faceted. This might result in a higher contact area with the substrate.

To simulate the effect of a higher contact area we not only simulated the Pd shell (3.5 nm) as a sphere (Supplementary Figure 12a) but also as an icosahedron with the same volume (V = $2.1*10^5$ nm^3; inradius of the icosahedron 34.755 nm). As the temperature differences within the NP (Au NS and Pd shell) are negligible, the entire NP is associated with the heat source. We neglect the effect of the facets on the absorption cross section and use the value for a 67 nm sphere with a 3.5 nm Pd shell as calculated using Lumerical FDTD ($\sigma_{\text{abs}} = 1.25 * 10^{-14}\ \text{m}^2$). We rotate the icosahedron to result in a point contact (Supplementary Figure 14b) and a facet contact (Supplementary Figure 14c) with the substrate and leave all other parameters unaltered. Supplementary Figure 14d shows the temperature profile from the center of the NP along x. The calculated temperature decrease due to the higher contact area is ≈4%. In this configuration, the resulting temperature of the faceted NS on the substrate is 84.3 % of the temperature of the NS in water.

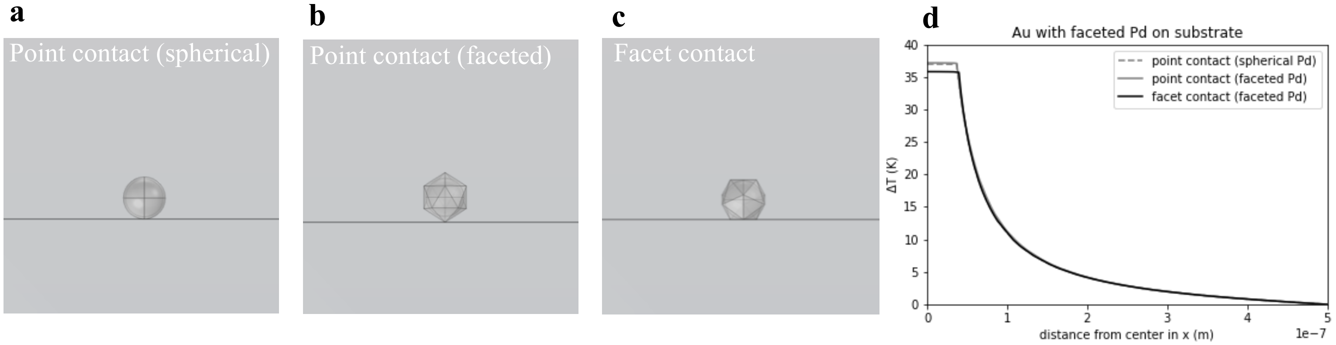

Supplementary Figure 14. A faceted Pd shell on a substrate simulated in COMSOL. The Pd shell is simulated as a (a) sphere and (b-c) an icosahedron. The icosahedron can be placed on the substrate with a single point of contact or a facet contact. (d) The temperature profiles from the center of the NPs in x direction.

<u>Supplementary Note 11. Characterization of Au60-Pd-sat NPs.</u>

The Au60-Pd-sat system is produced by solvent-induced electrostatic self-assembly between Au60 NS and Pd satellites. Supplementary Figure15 shows a size characterization of the Pd satellites, performed by Transmission Electron Microscopy (TEM).

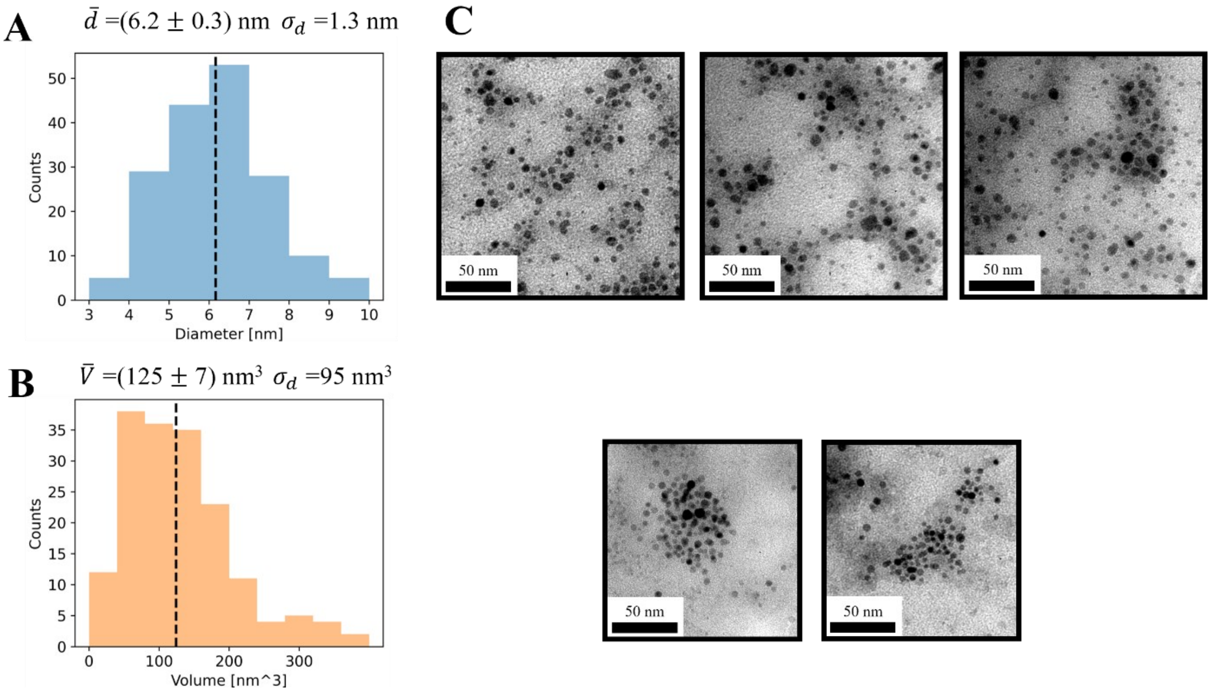

Supplementary Figure 15. Size characterization of Pd satellites. **(a)** Histogram of the diameter of Pd Satellites. The median diameter is d = (6.2 ± 0.3) nm with a standard deviation of σ_d =1.3 nm. **(b)** Histogram of the volume of Pd Satellites. The median volume of the Pd satellites was estimated to be V = (125 ± 7) nm^3 with a standard deviation of σ_V = 120 nm^3. **(c)** TEM images of the Pd satellites.

A TEM image of the successfully assembled of Au60-Pd-sat NPs is shown in Supplementary Figure 16.

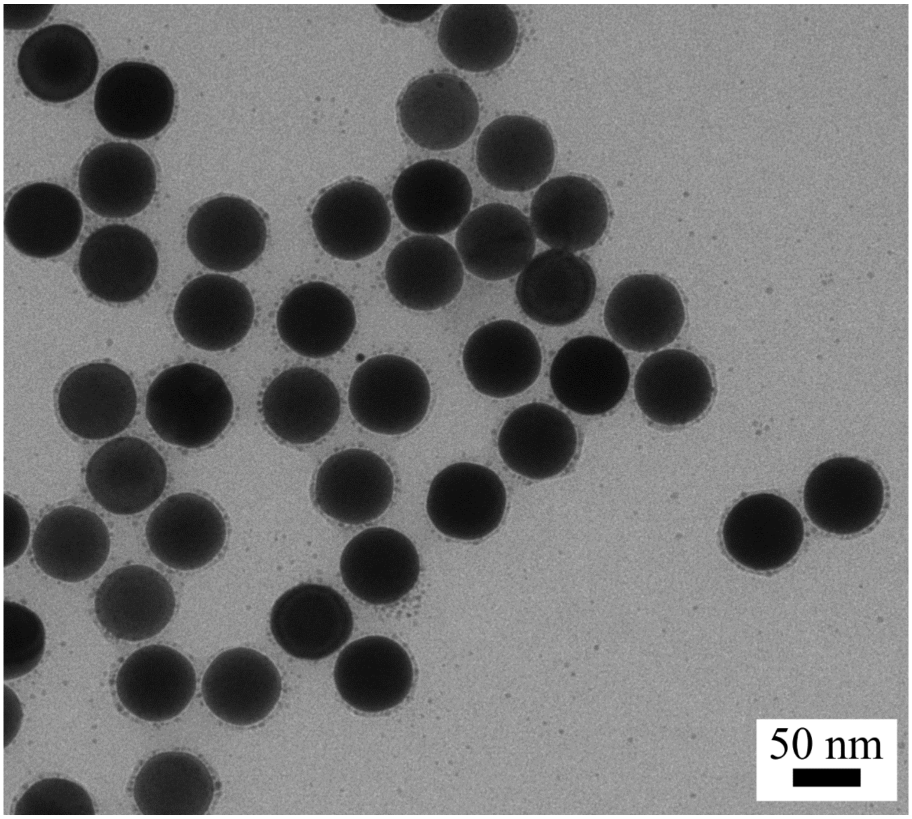

Supplementary Figure 16. TEM image of Au60-Pd satellites.

Supplementary Figure 17 shows a Scanning Transmission Electron Microscopy (STEM) in High Angle Annular Darkfield (HAADF) mode of a Au60-Pd-sat NP. The spatial distribution of the two metals measured by Energy Dispersive X-Ray Spectroscopy (EDX). The Au is mostly confined to the core, while Pd is only seen at the satellites.

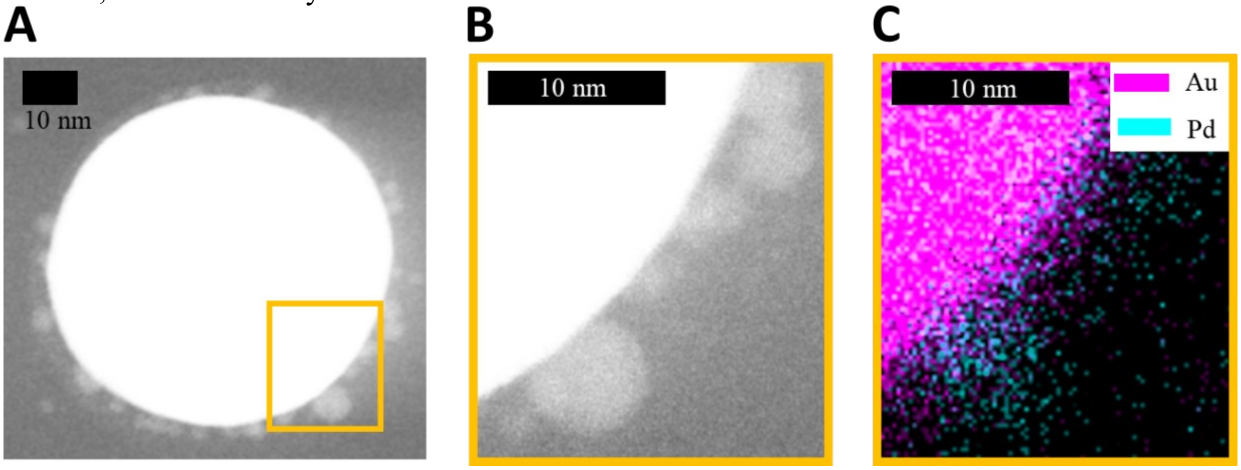

Supplementary Figure 17. EDX analysis of Au60-Pd satellites. (a) STEM-HAADF image of a Au60-Pd-sat NP. **(b)** Zoom in of the region marked in (a) with an orange square. **(c)** Spatial

distribution of Au and Pd in the same region is determined by EDX. Au (magenta) is mostly confined to the core, while Pd (cyan) is only seen at the satellites.
Supplementary Figure 18 shows a STEM image in Bright Field mode of a Au60-Pd-sat NP.

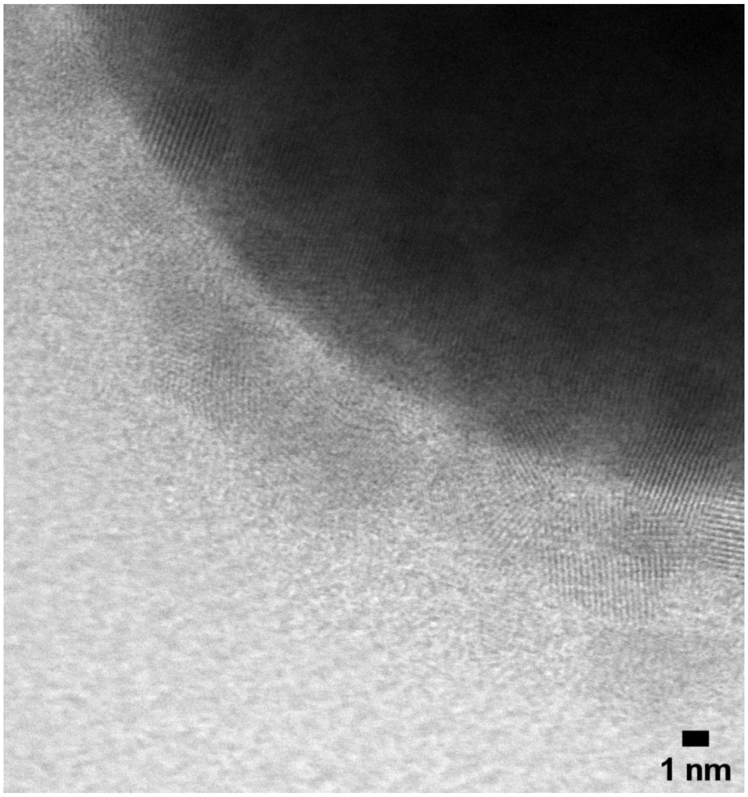

Supplementary Figure 18. STEM in Bright Field mode of a Au60-Pd-sat.

The number of satellites was estimated using ICP-AES.
As described in section S1, ICP-AES was used to determine the mass ratio between Pd and Au on this system, and a value $\frac{M_{\mathrm{Pd}}}{M_{\mathrm{Au}}}$ = (0.036 ± 0.002) was obtained, where M_{Pd} is the added mass of all the Pd satellites and $\mathrm{M_{Au}}$ is the mass of the Au core. See section Supplementary Note 15 for details on the ICP-AES characterization. The total volume of palladium per NP is $N_{\mathrm{s}}V_{\mathrm{sat}} = V_{\mathrm{C}}\frac{\rho_{\mathrm{Au}}}{\rho_{\mathrm{Pd}}}\frac{M_{\mathrm{Pd}}}{M_{\mathrm{Au}}}$, with V_{C} the volume of a Au core, V_{sat} the volume of a Pd satellite and N_{s} the number of satellites.

Assuming spherical shape of Au core with a diameter of (59.7 ± 0.2) nm (see Supplementary Figure 1) and $V_{\mathrm{sat}} = (125\ \pm 7)$ nm^3 (see Supplementary Figure 15). A value of $N_{\mathrm{s}} = (52 \pm\ 7)$ was obtained.

Supplementary Figure 19 shows the calculated absorption and scattering cross sections of a single 6.2 nm Pd nanosphere. The absorption and scattering cross sections at 532 nm are 10^4 and 10^6 times smaller than a 60 nm Au NS, respectively.

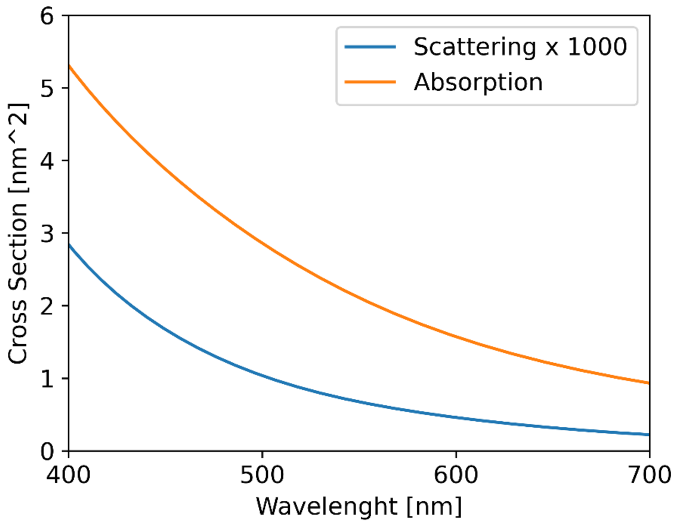

Supplementary Figure 19. Optical Properties of Pd satellites. Absorption (black) and scattering (red) cross sections of 6.2 nm Pd nanospheres. Scattering was multiplied by 1000.

Supplementary Note 12. The gap size between the palladium satellites and the gold surface.

The Au60-Pd-sat system is produced by solvent-induced electrostatic self-assembly between a positive CTAC capped Au NP and negative Polyvinylpyrrolidone (PVP) capped Pd satellites. Therefore, the gap between both surfaces is expected to be in the order of magnitude of the size of the capping molecules.

To confirm this, we have performed STEM-HAADF, as shown in Supplementary Figure 20. Due to the 3D geometry of the system, the satellites that are above or below the equatorial plane of the Au sphere appear like they are penetrating the Au core. For this reason, the gap was measured in the satellites that look further from the Au surface. The distance between surfaces is approximately 1 nm, which can be considered to be an upper bound. On the other hand, the fact that we observed a gap for the NPs in the equatorial plane suggest that such a gap exists in most cases, and that the contact surface between materials is negligible.

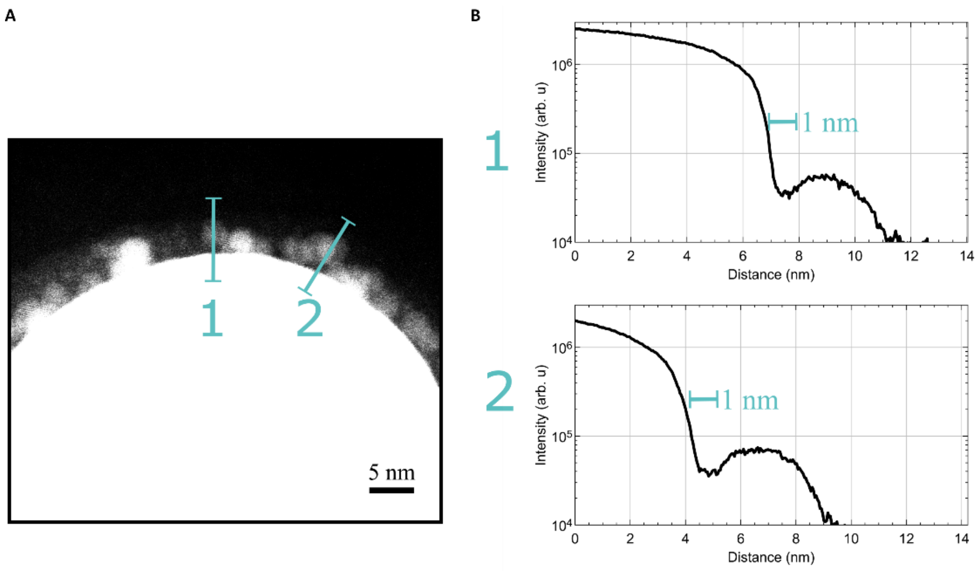

Supplementary Figure 20. Determination of gap sizes. (a) Scanning Transmission Electron Microscopy of a Au60-Pd satellites NP. The two profiles drawn in the figure are plot in (b).

To further discuss the impact of the gap size in the optical properties, we have calculated the absorption cross section of the system for different gap distances between the Au and the Pd satellites, as shown in Supplementary Figure 21. The total number is $N = 50$, the radius is 3.25 nm, the spatial distribution is approximately equidistant. This is done by arranging nodes along a spiral in such a way that the distance between nodes along the spiral is approximately equal to the distance between coils of the spiral. The arrangement of the satellites is shown in the inset of Supplementary Figure 21.

The change in absorption at 532 nm with respect to an Au core without satellites is -5.5%, -4%, and -0.5% for gap sizes of 0 nm, 0.5 nm, and 1 nm, respectively. These estimated reductions are consistent with the experimentally measured 2% reduction of the mean photothermal coefficient β between AuNS-60nm and Au60-Pd-sat.

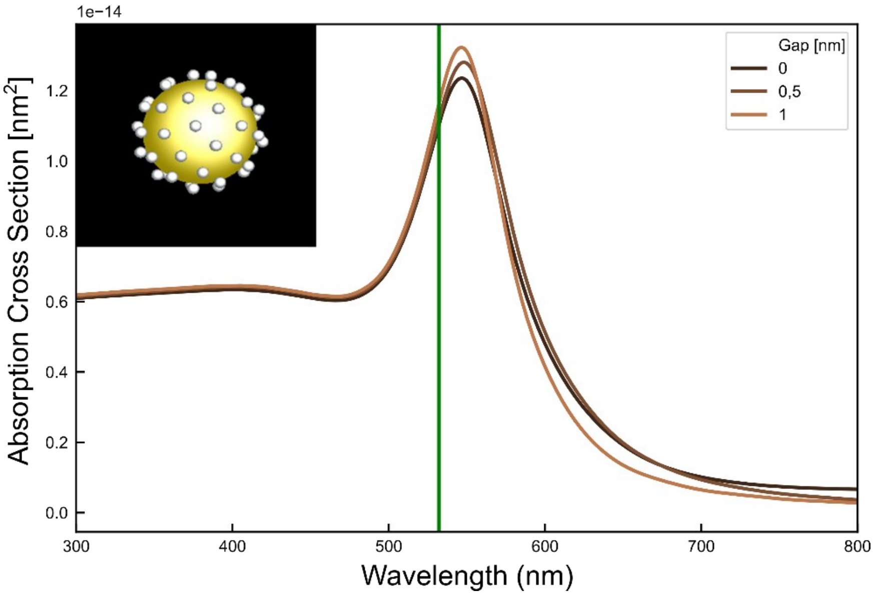

Supplementary Figure 21. Absorption cross section of a Au60-Pd satellites for different gap sizes between the satellites and the Au surface. The green line indicates the 532 nm laser. The inset shows the arrangement of the satellites used in the calculations.

Supplementary Note 13. Calculation of absorption efficiencies.

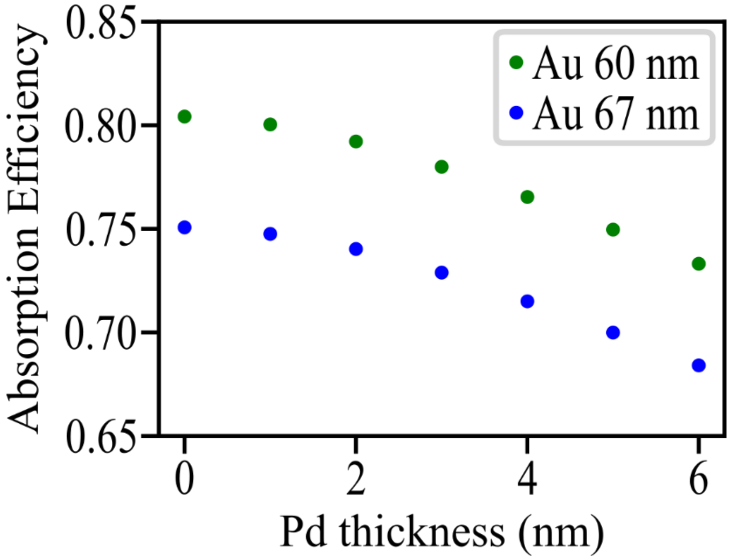

Supplementary Figure 22. Calculated Absorption Efficiencies at 532 nm. Green and blue dots correspond to Au@Pd Core-shell NPs with a core size of 60 nm and 67 nm respectively.

Supplementary Note 14. Photothermal response of alternative materials.

To predict the temperature of bimetallic core-shell nanostructures, we simulated the absorption of different Au@M (*M*=Pd, Pt, Rh, Ag) within the visible range. For the model, 60 nm Au spheres and shells of 2 nm in thickness were employed. The obtained absorption cross-sections (σ_{Abs}) are presented in Supplementary Figure 23A. The absorption cross-section at all wavelengths was used to determine the temperature increase. The calculations were conducted assuming that the role of the substrate was the same for all of the system (*f*=0.875) and that the term containing the interfacial resistance was negligible. The temperature increase as a function of the wavelength is shown in Supplementary Figure 23B.

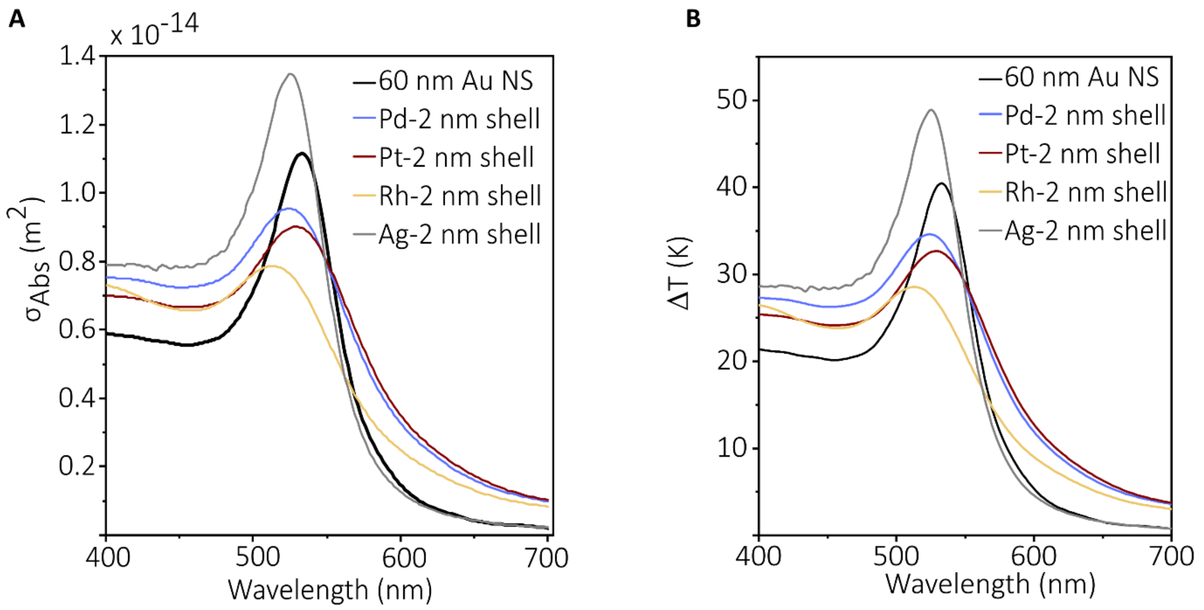

Supplementary Figure 23. Prediction of median temperature increase for Au@M core-shell structures (*M*=Pd, Pt, Rh, Ag). (a) Absorption cross-section (σ_{Abs}) for Au@M core-shell nanoparticles across the visible range. 60 nm Au NS were used as core, while the shell was 2 nm thick. **(b)** Predicted temperature increase for all systems as a function of wavelength, corresponding to an irradiance of 1 mW μm^{-2}.

Supplementary Note 15. Calibration for Inductively coupled plasma – atomic emission spectroscopy.

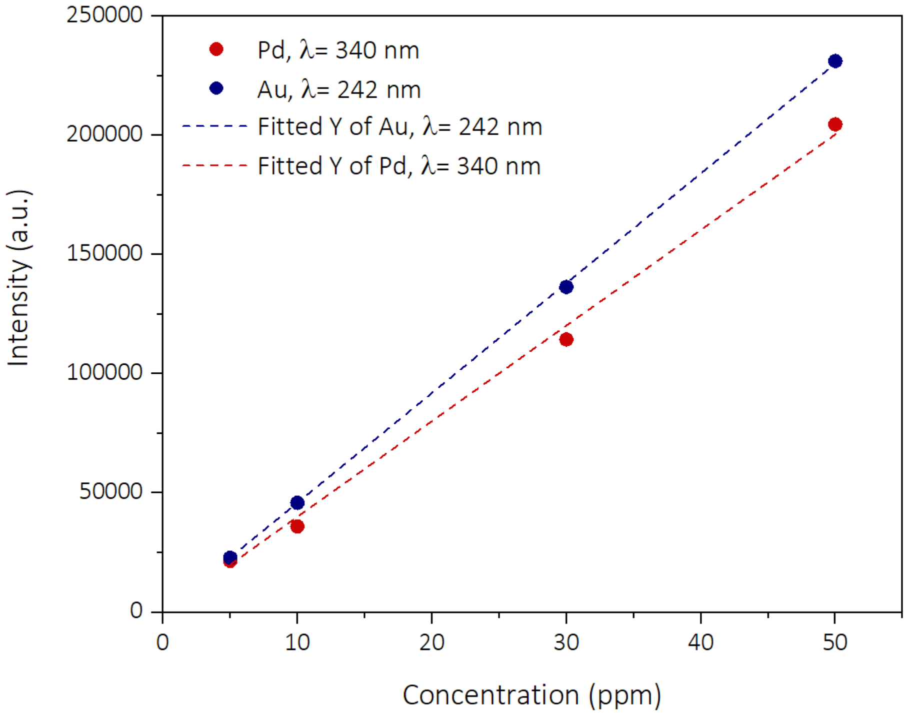

Supplementary Figure 24. Calibration curves for ICP-AES. The fitted values are: Au Concentration (ppm) = Intensity/4602,74. For Pd, Concentration (ppm) = Intensity/4009,53

Supplementary References

1. Gargiulo, J. *et al.* Accuracy and Mechanistic Details of Optical Printing of Single Au and Ag Nanoparticles. *ACS Nano* **11**, 9678–9688 (2017).
2. Gundrum, B. C., Cahill, D. G. & Averback, R. S. Thermal conductance of metal-metal interfaces. *Phys. Rev. B - Condens. Matter Mater. Phys.* **72**, 1–5 (2005).
3. Galinon, C. *et al.* Pd/Ag and Pd/Au interface specific resistances and interfacial spin flipping. *Appl. Phys. Lett.* **86**, 1–3 (2005).
4. Block, A. *et al.* Tracking ultrafast hot-electron diffusion in space and time by ultrafast thermomodulation microscopy. *Sci. Adv.* **5**, eaav8965 (2019).
5. Lin, Z., Zhigilei, L. V. & Celli, V. Electron-phonon coupling and electron heat capacity of metals under conditions of strong electron-phonon nonequilibrium. *Phys. Rev. B - Condens. Matter Mater. Phys.* **77**, 1–17 (2008).
6. Medvedev, N. & Milov, I. Electron-phonon coupling in metals at high electronic temperatures. *Phys. Rev. B* **102**, 1–22 (2020).
7. Wang, L., Sagaguchi, T., Okuhata, T., Tsuboi, M. & Tamai, N. Electron and Phonon Dynamics in Hexagonal Pd Nanosheets and Ag/Pd/Ag Sandwich Nanoplates. *ACS Nano* **11**, 1180–1188 (2017).
8. Wang, W. & Cahill, D. G. Limits to thermal transport in nanoscale metal bilayers due to weak electron-phonon coupling in Au and Cu. *Phys. Rev. Lett.* **109**, 1–5 (2012).
